# Predicting miRNA-based disease-disease relationships through network diffusion on multi-omics biological data

**DOI:** 10.1038/s41598-020-65633-6

**Published:** 2020-05-26

**Authors:** Marissa Sumathipala, Scott T. Weiss

**Affiliations:** 1 Channing Division of Network Medicine, Department of Medicine, Brigham and Women’s Hospital, Harvard Medical School, Boston, MA USA; 2 Department of Medicine, Harvard Medical School, Boston, MA USA; 3 Harvard College, Cambridge, MA USA

**Keywords:** Data integration, Network topology, Predictive medicine, Probabilistic data networks, Computational models

## Abstract

With critical roles in regulating gene expression, miRNAs are strongly implicated in the pathophysiology of many complex diseases. Experimental methods to determine disease related miRNAs are time consuming and costly. Computationally predicting miRNA-disease associations has potential applications in finding miRNA therapeutic pathways and in understanding the role of miRNAs in disease-disease relationships. In this study, we propose the MiRNA-disease Association Prediction (MAP) method, an *in-silico* method to predict and prioritize miRNA-disease associations. The MAP method applies a network diffusion approach, starting from the known disease genes in a heterogenous network constructed from miRNA-gene associations, protein-protein interactions, and gene-disease associations. Validation using experimental data on miRNA-disease associations demonstrated superior performance to two current state-of-the-art methods, with areas under the ROC curve all over 0.8 for four types of cancer. MAP is successfully applied to predict differential miRNA expression in four cancer types. Most strikingly, disease-disease relationships in terms of shared miRNAs revealed hidden disease subtyping comparable to that of previous work on shared genes between diseases, with applications for multi-omics characterization of disease relationships.

## Introduction

MiRNAs are small non-coding RNAs that regulate critical biological processes like cell proliferation, development and metabolism^1^. MiRNAs target the 3′UTR of genes and downregulate or silence their expression. With dysregulation of miRNAs having been reported in many diseases, it is not surprising that identifying and prioritizing disease associated miRNAs is an ongoing focus of research^2,3^. The ability of miRNAs to simultaneously target multiple genes makes them an attractive alternative to the ‘one target, one drug’ model, but also decreases their overall disease specificity and increases the likelihood of potential off target effects^4^. Prioritizing disease specific miRNAs early in the drug development process will be key to developing viable miRNA therapeutics. The current standard for identifying miRNAs involved in a disease process is miRNA expression profiling, a time consuming, labor-intensive and expensive experimental approach. Expression profiling, which compares expression levels of various miRNAs in diseased and healthy tissues, bases miRNA identification upon the magnitude of their differential expression^5^.

Prior work has attempted to computationally predict miRNA-disease associations, employing a variety of machine learning and network-based methods. An early model by Jiang *et al*. constructed a network between functionally related miRNAs by computing the overlap in gene targets; this was integrated with phenome data, using known miRNA-disease associations to infer new associations^6^. Several studies have similarly relied on computing miRNA similarity and disease similarity networks to infer new miRNA-disease associations from known ones. Fu and Peng *et al*. developed a deep ensemble model, DeepMDA, that computed disease similarity by combining lncRNA-disease and gene-disease associations, and miRNA similarity was computed from known miRNA-disease and miRNA-gene associations^7^. Features extracted from the similarity information with stacked autoencoders were fed into a three-layer neural network to infer miRNA-disease associations. Chen *et al*. and colleagues developed several methods based on integrating known miRNA-disease associations, miRNA functional similarity, disease semantic similarity, and Gaussian interaction profile kernel similarity for miRNAs and diseases^8–12^. These include PBMDA, which applies a depth first search algorithm to a graph of miRNA similarity, disease similarity, and known miRNA-disease associations; MDHGI, which applies matrix decomposition to known miRNA-disease associations, then constructs a heterogenous network with miRNA similarity, disease similarity, and miRNA-disease associations; BNPMDA, which uses a bipartite network recommendation algorithm for assigning transfer weights for links between miRNAs and diseases; and others. Gu et al. integrated data including known miRNA-disease associations, miRNA functional similarity, and disease semantic similarity to construct miRNA-miRNA and disease-disease similarity networks, which are then projected and combined to predict potential miRNA-disease associations^13^. The M2LFL method developed by Xiao *et al*. obtains similarity profiles from multiple data sources such as miRNA functional similarity, disease semantic similarity, and known miRNA-disease associations, from which latent geometric features are extracted and used to infer new miRNA-disease associations^14^. Several recent methods have been developed within the last year that also rely on miRNA and disease similarity networks to infer novel miRNA-disease associations, employing methods such as neural networks, computation of a Laplacian graph score, kernel fusion, and graph convolutional networks^15–18^.

Several studies have adopted network diffusion based methods. He et al. developed a method called CNMDA that applies a random walk with restart to a composite network constructed from known miRNA-disease associations; Gaussian interaction profile kernel similarity for long non coding RNAs (lncRNAs), miRNAs, and diseases; disease semantic similarity; miRNA functional similarity; known miRNA-lncRNA interactions; and known lncRNA-disease associations^19^. Yu *et al*. constructed a miRNA-disease transition probability matrix by applying a hybrid recommendation algorithm to known miRNA-disease associations^20^. This is followed by an unbalanced bi-random walk on a heterogenous network comprised of the miRNA-disease transition matrix, disease similarity matrix, and miRNA similarity matrix. Chen *et al*. applied a random walk with restart on a miRNA-miRNA functional similarity network^21^. These studies, and many others, rely on experimentally known miRNA-disease associations as input data to infer new miRNA-disease associations. Yet, because miRNA-disease association predictions are at an early stage, experimentally known associations are limited. Thus, predicting miRNA-disease associations without relying on known miRNA-disease associations is of importance.

Many of these prior methods rely on the assumption that functionally similar miRNAs will target phenotypically similar diseases, reflecting a longstanding hypothesis in the field. Yet, robustly validating this assumption remains an ongoing challenge. It is well established that phenotypically similar diseases share genetic origins, first synthesized into the disease module hypothesis of Barabasi *et al*.^22^ Indeed, Goh *et al*. discovered that in a network of disease nodes linked by their shared genes, disease classes such as cancers formed tightly interconnected clusters held together by a small set of shared genes, such as P53 and NF1 in the case of cancers^23^. Yet, whether the same phenomenon of diseases clustering by their common shared genes extends to miRNAs remains an unanswered question.

Network medicine is an emerging paradigm to better understand the molecular interactions underlying disease and elucidate disease-disease relationships^22^. Advances in network medicine have enabled the prioritization of traditional drug targets, such as small molecules and proteins, using disease-gene associations and protein-protein interactions^24,25^. While gene-disease networks and their emergent properties are well studied^23^, the mapping of miRNA-disease networks is still in its infancy. As miRNAs are part of a complex regulatory network where each miRNA regulates multiple genes and a gene is regulated by several miRNAs and other genes, it is important to account for both miRNA-gene associations and interactions between genes and their protein products^4^. In this study, we apply a network diffusion method that exploits the local neighborhood network connectivity among miRNAs and proteins to predict new miRNA-disease associations. Network diffusion methods are based on the small world property of gene networks, where protein products of genes associated with a disease have a strong tendency to interact with each other^26^. Such methods identify destination nodes, such as miRNAs, that are frequently reached from the seed nodes, such as disease genes. Destination nodes with high scores have redundant paths from the seed nodes, meaning that even if the network is missing edges because of incomplete data, the results would be similar. In contrast, methods that calculate the shortest path between the seed genes and destination nodes are highly sensitive to missing links. Several previous studies have successfully proposed network diffusion methods to analyze molecular interaction networks, called an interactome, even when the interactome data is incomplete. These studies apply the guilt by association principle to the interactome to predict lncRNA-disease associations, prioritize disease genes, predict biochemical perturbation patterns, pinpoint biomarkers, predict cancer mutations, and find network modules for specific diseases like asthma and COPD^27–33^. Santolini *et al*. found a network diffusion model of protein-protein interactions could recover the magnitude and direction of gene expression perturbations^33^. Sumathipala *et al*. applied a random walk with restart to subnetworks of the protein-protein interactome to predict lncRNA-disease associations^29^.

Here, we generalize the guilt by association principle to miRNA-related interactome data to reveal the role of miRNAs in complex disease mechanisms and in disease-disease relationships. This is in contrast to many previous studies in miRNA-disease prediction that apply a random walk to miRNA similarity, disease similarity networks, and known miRNA-disease associations, rather than to multi-level molecular interactome data.

In this study, we take a conceptually different approach to previous studies, proposing the **m**iRNA-disease **a**ssociation **p**rediction (MAP) method. MAP is a network diffusion method that integrates miRNA-gene, protein-protein, and gene-disease network information into a multi-level complex network to predict and prioritize biologically relevant miRNAs for diseases. We evaluate the performance of MAP using independent experimental miRNA-disease association and differential expression data. Second, we explore the emergent properties of our predicted miRNA-disease network by analyzing disease-disease relationships based on shared miRNAs for disease subtyping.

## Results

### Predicting the miRNA-disease network

**Figure 1:**
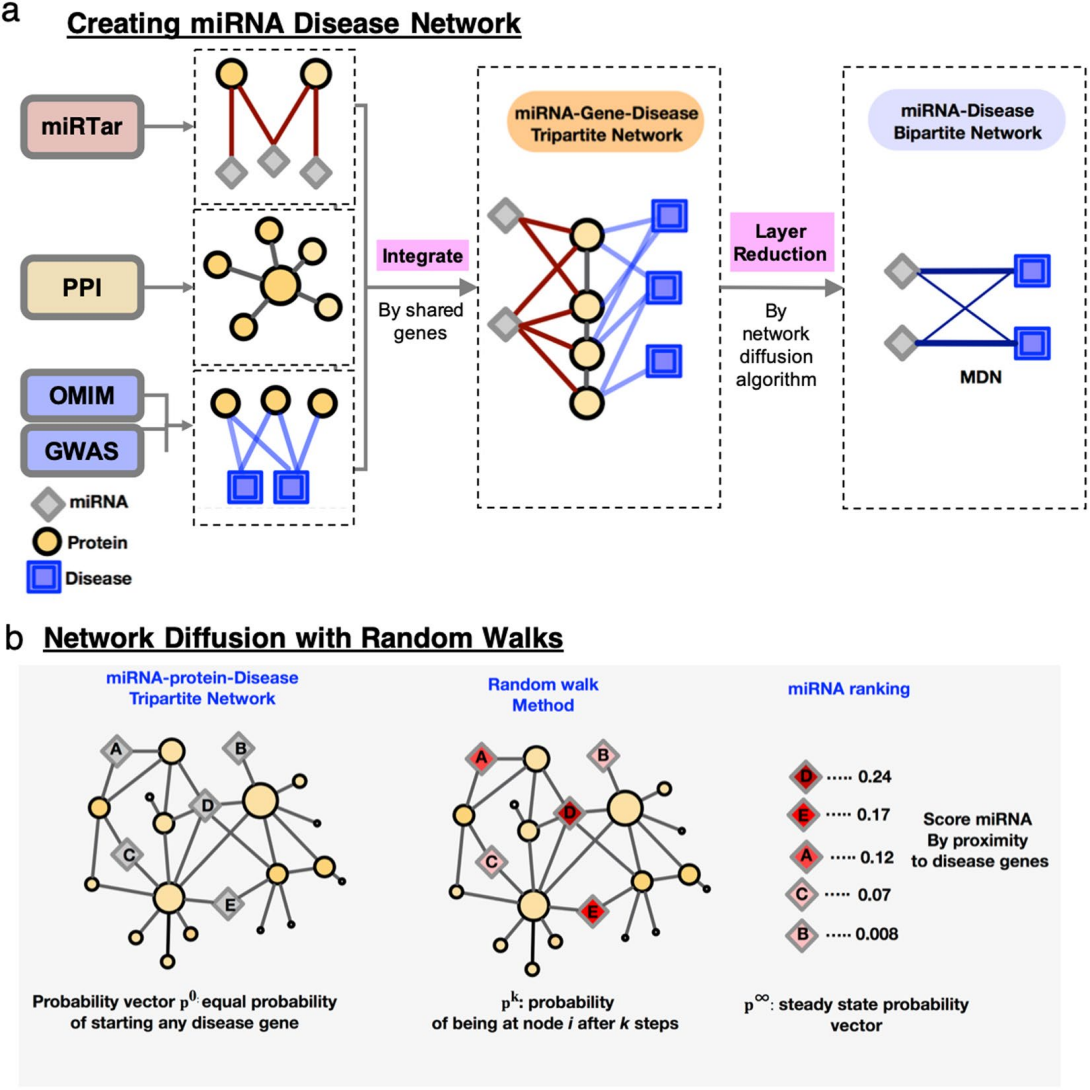
Workflow of MAP method for miRNA-disease association prediction. (**a**) The miRNA disease network is constructed by combining miRNA-gene associations from the miRTar database, protein-protein interactions (PPI), and gene-disease associations from OMIM and GWAS databases, to form a heterogeneous tripartite network. (**b**) Using a network diffusion algorithm that performs a random walk starting at the known disease genes to rank miRNA-disease associations, the tripartite network is reduced to form the final miRNA-disease bipartite network.

While many current methods rely on known miRNA-disease associations to infer novel associations, here we predict the miRNA-disease network without *a priori* miRNA-disease data. We combine 28,488 gene-disease associations from OMIM and GWAS databases^34^, 141,296 protein-protein interactions^24^, and 319,791 miRNA-gene interactions from the miRTar database to form a tripartite network (Fig. 1a)^35^. Next, we apply MAP to rank miRNA candidates for each disease by their proximity to known disease genes in the network (Fig. 1b). For each of the 4,454 diseases in our datasets, the MAP network diffusion algorithm ranks all 1777 miRNA candidates, yielding a bipartite miRNA-disease network where each weighted edge represents a predicted association between a disease and a miRNA. Each miRNA-disease association is weighted with the probability of the random walker moving from a known disease gene to a miRNA candidate.

### Experimental validation

We use experimental miRNA-disease associations from the dbDEMC database of differentially expressed miRNAs in cancer^36^ to validate MAP’s predicted miRNA-disease associations, as described in Methods. The dbDEMC database contains 34,952 interactions between 34 cancers and 1,467 miRNAs.

We evaluated the performance of MAP by computing area under the receiver operating characteristic curve (AUC). To generate the receiver operating characteristic (ROC) curves, we plot the false positive rate and true positive rate at different thresholds, using the predicted miRNA-disease edge weights as thresholds. A perfect classifier would yield an AUC of 1 while a random classifier would yield an AUC of 0.5. An AUC above 0.8 is considered good performance and above 0.9 is considered excellent performance.

To assess the relative performance of MAP, we computed ROC curves for four methods: (1) MAP method, (2) a current state-of-the-art method called CNMDA, (3) another classical method called MDHGI and (4) a randomized network model as a negative control. CNMDA applies a random walk with restart to a composite network formed from known miRNA-disease associations, miRNA functional similarity data, disease semantic similarity data, and data on lncRNAs, another type of non-coding RNA, to predict new miRNA-disease associations^19^. MDHGI applies matrix decomposition to an adjacency matrix of known miRNA-disease associations to find the lowest rank matrix, which is used to reconstruct a new adjacency matrix, followed by combination with miRNA similarity and disease similarity to form a heterogenous network^10^. Unlike MAP, CNMDA and MDHGI infer new miRNA-disease associations from known miRNA-disease associations, relying on the assumption that similar diseases will share functionally similar miRNAs. We generated the random network by shuffling edge weights on the miRNA-disease network predicted by MAP. The shuffling effectively randomizes the miRNA rankings, creating a null model as a control for comparison with MAP’s prediction.

**Figure 2:**
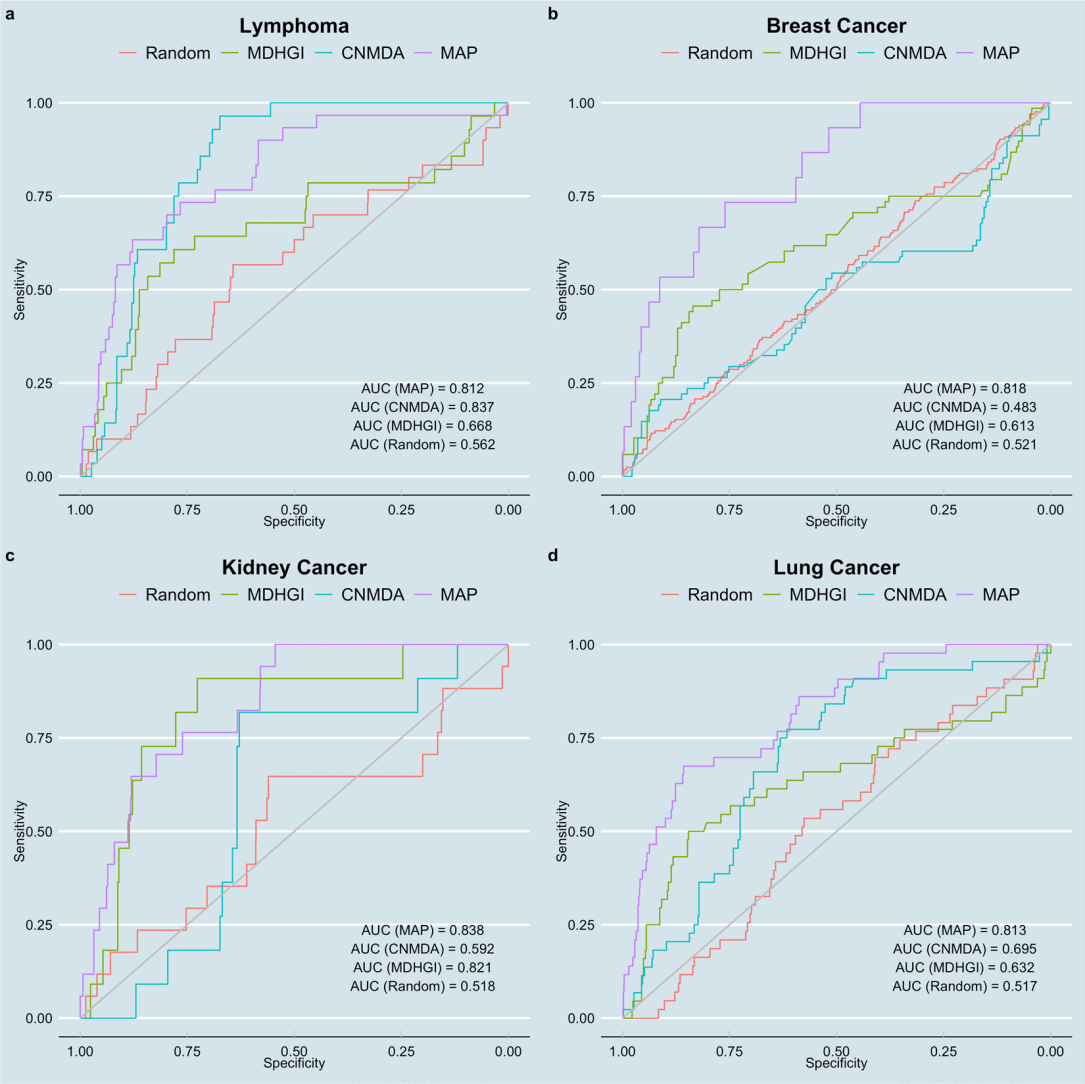
MAP’s performance in predicting miRNA disease associations for four cancers, evaluated using experimental miRNA-disease associations from the dbDEMC database. For each cancer, four ROC curves are shown: (1) MAP, (2) CNMDA, a current state of the art method for miRNA-disease association prediction, (3) MDHGI, another current method for miRNA-disease association prediction, and (4) a randomized network generated from node label shuffling, as a negative control.

Evaluated on four cancers, MAP yielded AUCs of 0.81, 0.82, 0.84, and 0.81 for lymphoma, breast cancer, kidney cancer and lung cancer, respectively (Fig. 2). MAP had strong performance in predicting miRNA-disease associations with AUCs all above 0.8. The null model predictions all had low AUCs, of around 0.5, confirming the predictor is making random guesses when the miRNA rankings are randomized. These findings indicate MAP is accurately predicting miRNA-disease associations by ranking miRNAs based on proximity to disease genes in the interactome.

CNMDA had AUCs of 0.837, 0.483, 0.592, and 0.695 for lymphoma, breast cancer, kidney cancer, and lung cancer, respectively. Compared to CNMDA, MAP outperforms CNMDA in breast cancer, kidney cancer, and lung cancer (DeLong’s test, p < 0.05), and has comparable performance in lymphoma (DeLong’s test, p > 0.05). MDHGI had AUCs of 0.67, 0.61, 0.82, and 0.63 for lymphoma, breast cancer, kidney cancer, and lung cancer, respectively. MAP outperforms MDHGI in lymphoma, breast cancer, and lung cancer (DeLong’s test, p < 0.05), and has comparable performance in kidney cancer (DeLong’s test, p > 0.05).

These findings confirm MAP’s ability to make predictions with equivalent or improved accuracy compared to CNMDA and MDHGI, current state-of-the-art methods. Of note, MAP does so without relying on *a priori* experimental miRNA-disease data.

### Predicting differential miRNA expression in cancer

Having validated the performance of MAP to predict miRNA-disease associations, we next assessed whether MAP could predict the strength of miRNA-disease associations. To do so, we assess if MAP can predict differential miRNA expression in cancer. We compared the edge weights predicted by MAP against the average fold change in miRNA expression, determined via clinical studies on patient cohorts with and without the cancer.

**Figure 3:**
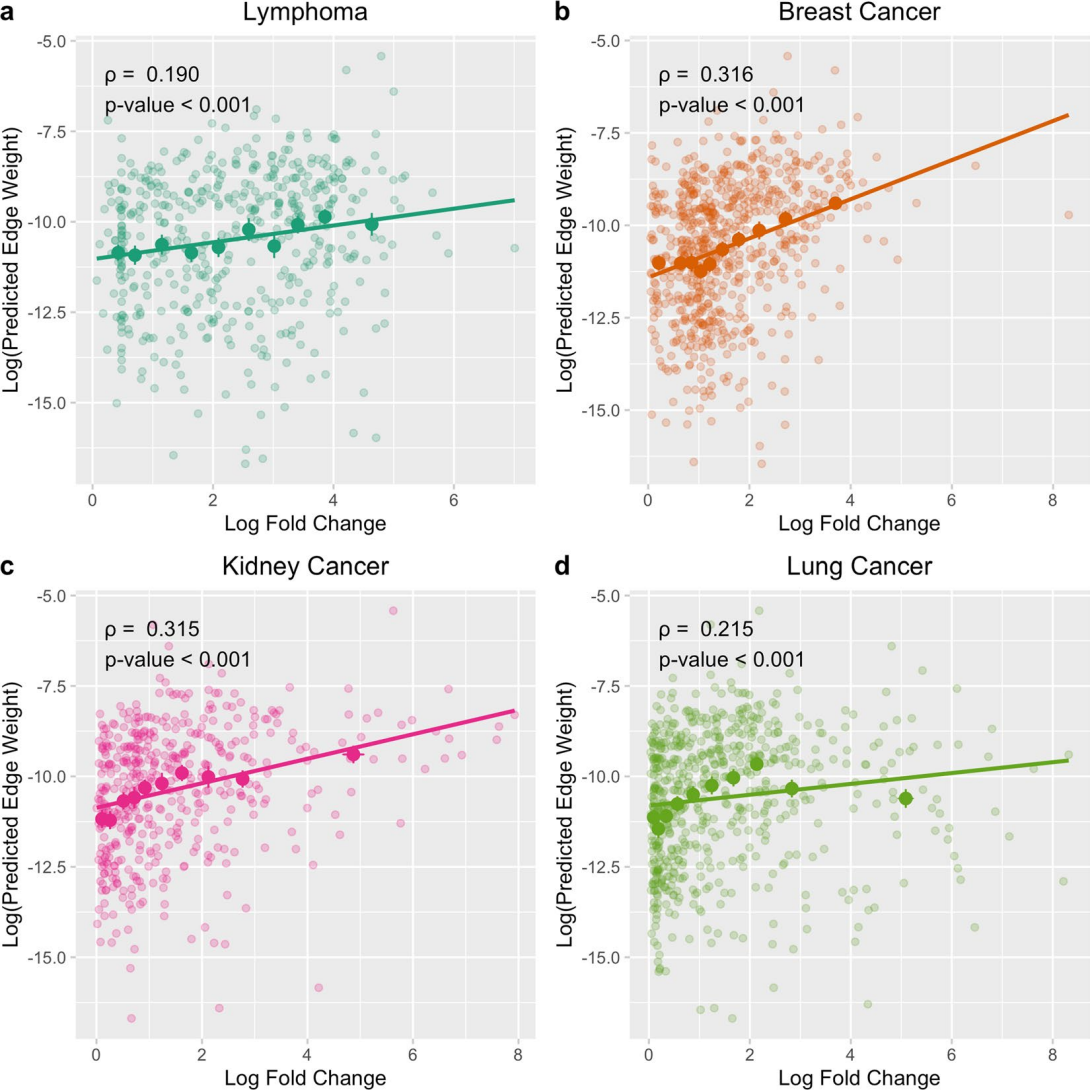
MAP predicts differential miRNA expression for four cancers. For each cancer, a scatterplot is shown where each small, translucent dot represents a miRNA associated with the cancer. The y axis value is the miRNA-disease edge weight predicted by MAP and the x axis value is the experimental fold change in miRNA expression between healthy and diseased patient cohorts, from the dbDEMC database. The large, solid dots represent the binned means and standard deviations, binned across the x axis. Spearman’s rank correlation coefficients and associated p-values are shown on the plot. Slope of linear regression models were significant for all four cancers (t-test, p < 0.05).

We found strong, positive correlations between the experimental differential miRNA expression and MAP’s predicted edge weights for lymphoma, breast cancer, kidney cancer, and lung cancer (Fig. 3). Spearman correlations between predicted edge weight and fold change were greater than 0.15 and highly statistically significant (p < 0.001) for all four cancers. The significant, positive Spearman correlation values demonstrate that the miRNAs with a large expression change in a cancer, meaning they are highly disease associated, tend to have a higher edge weight ranking with our MAP method. Further confirming these findings, we find a statistically significant linear relationship between known fold change in miRNA expression and predicted edge weight for all four cancers (t-test on slope of linear regression model, p < 0.05 for all four slopes). These findings demonstrate MAP’s ability to accurately prioritize highly disease associated miRNAs by predicting differential miRNA expression in disease.

### Disease-disease relationships in the predicted miRNA-disease network

Having validated our miRNA-disease network with experimental data, and directly compared it to state-of-the art methods, next we utilize our miRNA-disease network to study disease-disease relationships based on shared miRNAs. Prior work has analyzed disease-disease relationships based on shared genes. Goh *et al*. generated a disease projection in gene space, where nodes are diseases and two diseases are connected if they share one or more genes^23^. Because similar diseases have a strong tendency to share genes with each another, phenotypically similar diseases formed tightly interconnected communities in the disease interactome, enabling disease subtyping.

We investigated whether a disease projection in miRNA space would exhibit similar structural communities of closely related diseases. We created a disease projection in miRNA space (DPM) from the bipartite miRNA-disease network predicted by MAP, as described in Methods. For this, we first thresholded the bipartite miRNA-disease network using the edge weights. Then we created a disease projection, which was thresholded again to yield the final DPM, in which two diseases are connected if they share one or more miRNAs. As a point of comparison for our DPM, we generate an analogous disease projection in gene space (DPG) to determine if the previously described clustering of similar diseases occurs in miRNA space.

We classified all disease nodes in the DPM and DPG into one of 14 broad disease classes, using the ICD9 standard medical code hierarchy, and visualized the two networks. To assess how structural communities of disease nodes correspond to the ICD9 disease classes, network visualizations are laid out based only on topology. The location of a disease node on the network visualization is determined by its connectivity to other disease nodes and is independent of its ICD9 class: enabling visual assessment of disease clustering by type.

**Figure 4:**
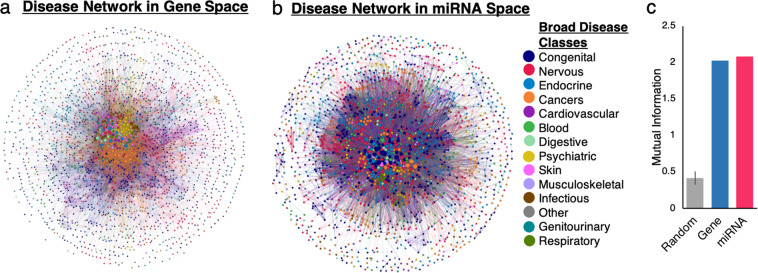
Disease projections show hidden disease-disease relationships in gene-disease and miRNA-disease bipartite networks. Each node is a disease and two nodes are connected if they are share genes or miRNAs. Link width represents number of shared genes or miRNAs. Node diameter represents number of neighboring disease nodes. Node color represents a broad disease class, assigned using ICD-9 medical codes. Network lay-out is based on topology and is independent of disease class. (**a**) Disease projection generated from bipartite gene-disease network. (**b**) Disease projection generated from bipartite miRNA-disease network predicted by MAP. (**c**) Mutual information between the ICD-9 metadata and structural community in each network is calculated for the (1) disease projection in gene space, (2) the disease projection in miRNA space, and (3) for 100 randomized networks as a negative control. Mutual information quantifies the extent to which diseases of the same broad disease class occupy the same structural community in the disease projection.

In gene space, diseases of the same broad class form distinguishable clusters, consistent with previous findings by Goh *et al*. (Fig. 4a). For example, in the center of the DPG, cancers (orange) and psychiatric disorders (yellow) form two visually distinguishable clusters. A similar degree of clustering is observed in miRNA space with homogenous clustering by disease type (Fig. 4b). For example, while cancers do not form a single orange cluster as in the DPG, we observed several smaller clusters of cancers (orange nodes) in the DPM. The clustering by disease class in the DPM results from phenotypically similar diseases being regulated by the same miRNAs.

To quantify the disease clustering we qualitatively observed in visualizations of the DPM and DPG, we use mutual information. Mutual information measures the alignment of topological clusters with disease subtype metadata by quantifying the amount of information shared between the two variables. Structural communities in the DPM and DPG are identified using the Louvain modularity algorithm^37^ and are independent of ICD9 disease class. As a negative control, we generate 100 randomized miRNA-disease networks with node label shuffling, from which we generate 100 randomized disease projections. We calculate average mutual information across the 100 randomized projections.

In the random networks, mutual information was only 0.42 with a standard deviation of 0.09. In contrast, mutual information for disease projections in miRNA space and in gene space were 2.10 and 2.03, respectively, over 4-fold larger (Fig. 4c). These findings provide quantitative support for the disease clustering by ICD9 class seen visually in Fig. 4a,b. Most importantly, mutual information in miRNA space was comparable to that in gene space. These findings demonstrate for the first time that at a broad scale view of disease-disease relationships, similar diseases form topological clusters based on their shared miRNAs. In other words, clinically related diseases share more miRNAs with each other than with dissimilar diseases, indicating there are miRNAs specific to certain disease classes like cancers.

### Subtyping from disease-disease relationships

We next investigated whether the trend of disease clustering by broad type (e.g. cancer, neurological diseases) extends to more specific disease subtypes: for instance, differentiating communities of specific cancer types, such as bone and bladder cancers. For this, we generated subnetworks of the DPM and DPG. Each subnetwork is comprised of all diseases in a particular broad class: in each, nodes are colored by disease subtype, such as blood cancers, as determined by ICD9 medical code hierarchy. We generated subnetworks for cancers, neurological diseases and cardiovascular diseases.

**Figure 5:**
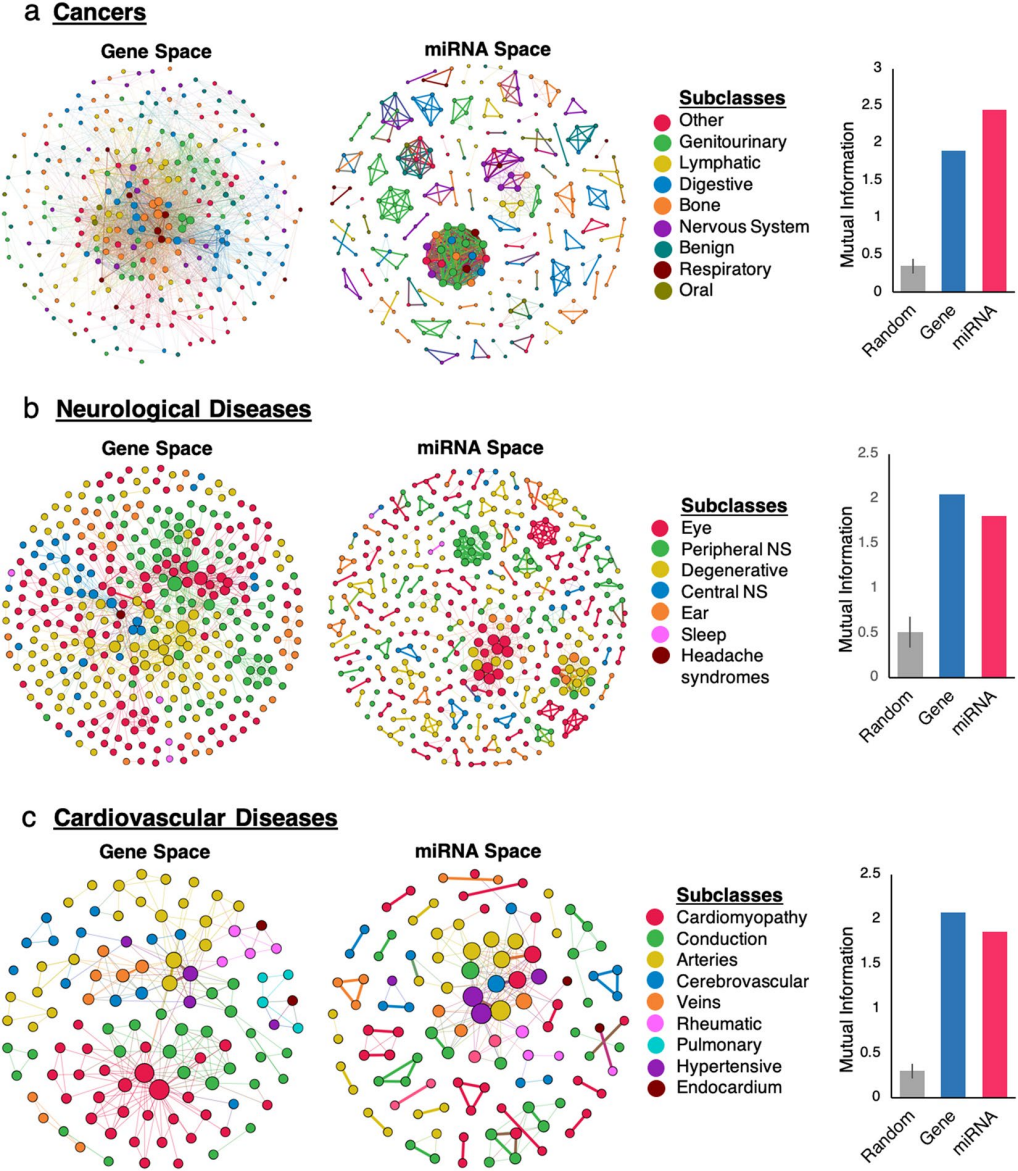
Subnetworks of disease projections show clustering by specific disease subtypes. Subnetworks are generated by filtering all the disease nodes within a broad disease class. Node color represents a specific disease subtype within the broad class, assigned using ICD-9 medical codes. Each node is a disease and two nodes are connected if they are share genes or miRNAs. Link width represents number of shared genes or miRNAs. Node diameter represents number of neighboring disease nodes. Network lay-out is based on topology and is independent of disease class. (**a**) Subnetworks of all disease-disease relationships for cancers, in both miRNA and gene space, and mutual information to quantify clustering of cancers by subtype. (**b**) Subnetworks of all disease-disease relationships for neurological diseases and mutual information. (**c**) Subnetwork of all disease-disease relationships for cardiovascular diseases, and mutual information.

Similar to the full disease projections in Fig. 4, the cancer subnetwork in gene space has some topological separation of cancer subtypes (Fig. 5a). Cancers in miRNA space form even more separable structural communities of phenotypically similar cancers. This is further corroborated by mutual information values of 1.90 and 2.44 for gene space and miRNA space, respectively, both over fivefold higher than the random network (0.35 ± 0.09).

While in gene space the cancers are more densely connected than in miRNA space, many of the communities in miRNA space are highly separable, homogenous islands. These islands are groups of closely related cancers that share miRNAs with each other, but not pathologically distinct cancers. For example, there is a clique of seven bladder cancers (green) that don’t share high edge weight miRNAs with other cancers, yielding an island cluster (Fig. 5a). As can be seen visually, bladder cancers (green) in the gene space subnetwork do not form an analogous cluster but instead are more widely distributed across the network (Fig. 5a). The top-ranking miRNAs for these seven bladder cancers represent possible drug targets for treatment as they may be specific to bladder cancer. Overall, the findings in Fig. 5a demonstrate accurate and biologically relevant cancer subtyping can be derived from MAP’s predicted miRNA associations, corroborating the experimental validation with miRNA-cancer data (Figs. 2 and 3).

Next, we show that our miRNA-disease network can be applied to distinguish disease subtypes other than cancer. We create disease projection subnetworks for neurological diseases and cardiovascular diseases and find similar results (Fig. 5b,c). For all three of these subnetworks, there is above random clustering by disease subtype in miRNA space and in gene space, quantified by mutual information values that are several fold higher than random. The level of disease clustering by subtype in miRNA space is comparable to that in gene space for cancers, neurological and cardiovascular diseases, supported by similar mutual information values. This evidence of structural communities aligning with ICD9 subtype data for not only cancers, but also neurological diseases and cardiovascular diseases, supports the versatility of our network-based method for disease subtyping.

The high degree of clustering by disease type in the DPM holds true on both the full network with broad disease classification (Fig. 5) and on subgraphs with narrower disease classification (Fig. 5), corroborating the ability of our method for disease subtyping using miRNAs.

## Discussion

In this work we build on the success of prior work applying network methods to interactome data to develop MAP, a network medicine framework to predict miRNA-disease associations and elucidate disease-disease relationships. We combine massive genomic and transcriptomic datasets into a miRNA-gene-disease tripartite network and apply a network diffusion algorithm to rank miRNA candidates for a disease, producing a miRNA-disease bipartite network. MAP powerfully predicts miRNA-disease associations with its unique method of applying network diffusion to multi-omics data. This is in contrast to many prior studies that used a single omics type of data, such as the protein-protein interactome, and sets a precedent for further work in extracting information from multi-level networks constructed by integrating varied omics data.

Many current methods for predicting miRNA-disease associations utilize experimentally known associations to infer novel miRNA-disease links. In contrast, MAP exploits the local neighborhood property of the complex multilevel network we construct to rank miRNAs based on their proximity to known disease genes. As a result, MAP can be applied to predict and prioritize miRNAs for any disease using its known disease genes.

Despite not using *a priori* knowledge of miRNA-disease associations, MAP retains high performance that is comparable or superior to current state-of-the-art methods such as CNMDA and MDHGI^10,19^. When compared to experimental miRNA-cancer association data, MAP accurately predicted miRNA-disease associations for lymphoma, breast cancer, kidney cancer, and lung cancer, with AUCs all over 0.8. When compared with the MDHGI method, MAP had improved performance for breast cancer, lung cancer, and lymphoma, and comparable performance for kidney cancer. Moreover, when compared with the performance of the CNMDA method, MAP had improved performance in predicting miRNAs for breast cancer, lung cancer, and kidney cancer and comparable performance for lymphoma. While both CNMDA and MAP utilize a random walk with restart, the starting networks vary greatly. CNMDA relies on using known miRNA-disease associations to infer new ones, and does not utilize protein or gene interaction data. In contrast, MAP’s predictions are independent of known miRNA-disease associations, instead relying on the combined topology of the protein interactome and miRNA-gene associations. As MAP only requires a set of known genes, it is more easily generalized to new contexts, such as diseases with no known miRNAs or predicting patient-specific miRNAs.

Previous methods, such as CNMDA and MDHGI, have predicted binary disease association for miRNAs, but to date, no studies have predicted differential miRNA expression. Motivated by the success of prior work predicting gene expression using network topology^38^, we compare MAP’s predicted miRNA-disease edge weights to experimental data on differential miRNA expression in cancers. Strong positive correlations demonstrate MAP accurately predicts differential miRNA expression using only topology of the protein and the miRNA interactome: to our knowledge, MAP is the first such method to predict differential miRNA expression. As miRNA drug targets are traditionally found via differential expression profiling, which are costly and time consuming, network based prediction of differential expression has the potential to streamline miRNA drug target discovery.

In addition to differential expression prediction, the miRNA-disease bipartite network predicted by MAP can uncover disease-disease relationships and disease subtyping. For this, we generated a network of disease-disease relationships based on MAP’s predictions of shared miRNAs. We find diseases naturally separate into clusters of phenotypically similar diseases that share top ranking miRNAs. When compared to an analogous disease-disease network based on shared genes, we find a comparable level of clustering, quantified with similar values of mutual information. For example, in both the miRNA space disease network and gene space disease network, cancers aggregate into tightly connected, distinguishable clusters.

In further fine-grained analysis of this disease subtyping through a subnetwork of all cancers, we find our miRNA-disease network can not only cluster diseases by broad types like cancer, but by subtypes of cancer, such as bone cancers and genitourinary cancers. Representing disease-disease relationships with miRNAs separates cancers into distinguishable, homogenous clusters of phenotypically similar cancers.

This miRNA-based framework for disease relationships is not limited to cancers. When applied to cardiovascular and neurological diseases, diseases are distinguished by subtype. In addition to visual evidence of disease subtyping in the form of distinct, homogenous clusters in disease projection and subnetwork graphs, we find quantitative evidence for miRNA-based disease subtyping: mutual information between disease class and community was significantly higher than in a random network and higher than in gene space. Our findings of disease clustering suggest that the top ranked miRNAs are specific to a disease subtype. Further study of subtype-specific miRNAs may allow for deeper understanding of the molecular underpinnings of disease and could lead to more precise diagnoses and treatments.

It has been previously established by Goh *et al*. that in a disease-disease network based on shared genes the diseases will cluster by type^23^. However, to our knowledge, this study is the first to suggest that diseases can also cluster by their shared miRNAs. Importantly, this finding extends an underlying principle in network medicine—phenotypically similar diseases share close or overlapping disease modules in the protein interactome—to multi-omics data. The results of our disease-disease network analysis suggest that similar diseases share not only proteins, but also miRNAs, warranting further investigation into how non-coding RNAs, proteins, genes, and other biological components interact in a multi-level, complex network to influence disease-disease relationships.

Despite its promise, the limitations of MAP’s predictions include incompleteness of the protein-protein interactome. Previous work by Menche *et al*. demonstrated that accurate disease-disease relationships can be uncovered even in an incomplete protein-protein interactome^24^. The miRNA-gene association dataset may also be incomplete, especially given that miRNAs were discovered recently, and much work remains in systematically characterizing their regulatory roles. Moreover, the miRNA-gene association dataset, which is curated from the literature, may be subject to literature biases that could skew the dataset towards well studied miRNAs and genes. Another limitation is that the protein-protein interactome is not tissue specific; integrating data on tissue specific expression could improve MAP’s predictions. A third limitation is that the gene-disease associations for determining the random walker’s starting positions are unweighted; prioritizing crucial disease genes using differential gene expression could improve MAP’s predictions.

With growing evidence implicating miRNAs in complex disease, predicting novel miRNAs for diseases has significant biomedical applications, from studying the molecular underpinnings of disease, identifying new biomarkers, developing new therapeutics, and elucidating disease-disease relationships. Our MAP method not only accurately predicts miRNA-disease relationships, but also is successfully applied to uncover disease subtyping.

## Methods

### Constructing a multi-level complex network

The heterogeneous tripartite network was constructed by combining data from three sources:

#### MiRNA-gene network

We downloaded the miRTarBase dataset, which contains 422,517 experimentally validated miRNA-gene interactions between 4,076 miRNAs and 23,054 target genes^39^. The miRNA-gene interactions are extracted and manually curated from experimental studies in the literature. It contains the largest amount of validated miRNA-gene interactions, compared to similar miRNA-gene datasets.

#### Protein-protein interaction network

We use a protein-protein interaction dataset that combines interactions from sources such as kinase-substrate pairs, signaling interactions, literature curated interactions, and yeast two-hybrid datasets, to yield a network of 15,949 proteins with 217,140 interactions, as previously described^24^.

#### Disease-gene network

We downloaded from DisGenNET (www.disgenet.org) a publicly available dataset of gene-disease associations, and selected associations sourced from OMIM and GWAS datasets^40^. The dataset contained 28,488 associations between 6,464 protein-coding genes and 4,454 diseases.

### Network diffusion algorithm

After constructing the tripartite miRNA-gene-disease network by combining these three datasets, we use a random walk with restart to rank miRNAs for each disease (Fig. 1b). The random walk with restart algorithm ranks miRNAs based on proximity in the tripartite network to a disease’s known genes. The motivation of our approach lies in the local impact hypothesis, wherein cellular components linked to closely related diseases tend to interact with each other and form a localized network neighborhood.

The idea of the random walk with restart in MAP is that a walker begins at the set of known disease genes (seed genes) and moves by a series of random steps to other genes and miRNAs in the multi-level network, traveling via the protein-protein interaction and miRNA-gene edges. With each step from the current node to a neighboring node, a distribution p^k^ which represents the probability that the walker is at a given node at step *k*, is recalculated. At each step, the walker has a probability *a* = 0.7 of restarting at the seed genes.

Due to the large size of the tripartite network, simulating the random walk with restart for each of the 4,454 diseases would be highly computationally intensive. Instead, we adopt an analytical approach. We pre-calculate the converged matrix *P* for a random walk with restart, using the previously described equation for network propagation^26^:

$$P=a{(I-(1-a)W)}^{-1}$$

where *W* is the column normalized graph adjacency matrix, *a* is the restart probability, and *I* is the identity matrix.

For each disease, we create a vector *x* that represents the starting positions of the walker at the known disease genes. The walker has an equal probability of starting at any of the disease genes. We next multiply *x* by *P* to calculate an approximation of the steady state probability value, *p*^*∞*^. The *i*-th element of the steady state probability vector represents the probability of the walker moving from the seed genes to node *i*. The 1777 miRNAs are ranked for each disease according to their values in *p*^*∞*^.

$${p}^{\infty }=P\ast x$$

To validate our direct calculation of the converged matrix on an example disease, we calculated miRNA rankings for breast cancer using the more computationally intensive iterative method, using the same vector *x*. The Spearman correlation between the analytical method’s and the iterative method’s rankings was 0.98, confirming that the analytical method is indeed a very accurate approximation.

We repeat the matrix multiplication for all 4,464 diseases in the tripartite network, yielding the final weighted miRNA-disease bipartite network (MDN) where each disease has a set of 1777 ranked miRNA candidates. Each link in the network represents a predicted association between a miRNA and a disease, where the edge weight is the probability value from *p*^*∞*^. Pseudocode for the network diffusion algorithm is included in the Supplementary Information.

### Experimental validation of MAP using dbDEMC

To validate the miRNA-disease associations predicted by MAP, we use experimental miRNA-disease associations in the database on differentially expressed miRNAs in cancer (dbDEMC). The dbDEMC database has 34,592 experimentally validated miRNA-disease associations between 1,467 miRNAs and 34 cancers.

The miRNA-disease predictions were validated against the experimental dataset dbDEMC on four select cancers: lymphoma, breast cancer, lung cancer, and kidney cancer. For each cancer, we generate a receiver operating characteristic (ROC) curve and compute area under the ROC curve (AUC) using the dbDEMC associations as ground truths and the predicted edge weights from MAP. We compare MAP’s performance to the CNMDA and the MDHGI method to assess if MAP performs as well as current methods. With the same dbDEMC dataset, we also generate ROC curves and compute AUCs for the CNMDA method, the MDHGI method, and for the randomized null model network. We generate the randomized network by shuffling node labels on the bipartite miRNA-disease network.

Each miRNA-disease association in the dbDEMC database has a differential expression value that is calculated by comparing miRNA expression in healthy and diseased patient cohorts. Typically, miRNA drug targets are discovered by such expression profiling studies and selecting the miRNAs which are most differentially expressed (either over- or under-expression) for further drug development. Motivated by the high costs of expression profiling studies, we used the experimental differential expression data in dbDEMC to investigate if MAP could be used for in silico prediction of miRNA differential expression in cancer. We compared the edge weights predicted by MAP to experimental differential expression values from dbDEMC and used a Spearman correlation test to assess significance of the observed positive correlation. To assess the significance of the linear regression model fitted to the data, we performed a t-test on the slope of the linear regression analysis.

### Network analysis

#### Thresholding the bipartite miRNA-disease network

Our network diffusion approach ranks all 1777 miRNA candidates for every disease, but this yields a fully connected miRNA-disease bipartite network (MDN) with a graph density of 1. To shed light on the hidden network topology and elucidate disease-disease relationships, we must first threshold the MDN to filter important miRNA-disease associations. As we are interested in comparing disease projections of the MDN to that of the gene-disease network (GDN), we identify an edge weight threshold by comparing graph density in the MDN and GDN. We find a threshold of 0.001, where only edges with a weight greater than 0.001 are retained, reduces the graph density of the MDN to 0.0049. This is comparable to the graph density of the GDN, which is 0.00048.

#### Disease projection

To examine disease-disease relationships at a network level, we create a disease projection from the thresholded MDN; nodes are diseases and two diseases are connected if they have a predicted association with the same miRNA. The weight of each edge represents the number of miRNAs the two disease nodes share. For this, we use the igraph R package^41^. The weight of each edge is the number of shared miRNAs. Similarly, using the gene-disease association dataset from DisGenNet, we create an analogous disease projection in gene space (DPG), where two disease nodes are connected if they are linked to the same gene.

To threshold the disease projection in miRNA space, we again identify an edge weight cutoff that will reduce the graph density to a value comparable to the disease projection in gene space. We find a threshold of 7 reduces DPM’s graph density to 0.03, close to the DPG’s graph density of 0.005. We remove all nodes in the disease projections with a degree of 0.

#### Classifying diseases with ICD-9 standardized medical codes

To analyze if closely related diseases form clusters, disease nodes in the DPM and DPG were classified into categories using standardized International Classification of Disease 9 (ICD-9) medical codes. For converting disease names from the string format in the DisGenNet data source to ICD-9 medical codes, we use a classification file^42^. Using these medical codes, we first group all 4,464 diseases into one of 14 broad categories in the ICD-9, such as cancers, endocrine diseases, and neurological diseases. We next sub-group these categories using the next level of the ICD-9 hierarchy: for example, cancers are further classified into brain, bone, lung, etc. cancers.

#### Visualization

All network visualizations were generated using Gephi software (version 0.9.2). Networks were laid out using the Fruchterman Reingold Algorithm, a force directed layout algorithm in which nodes are treated as ‘atomic particles’ that exert attractive and repulsive forces from one another to better visualize clustering^43^. These forces are dependent upon the degree of the nodes; nodes with higher degrees (stronger attractive forces) are placed closer together and *vice versa*. The width of an edge on the visualization is proportional to its weight and the diameter of a node is proportional to its degree. Node color corresponds to the ICD-9 class.

#### Mutual information

The disease projections in miRNA space and gene space were analyzed to quantify the extent to which diseases of the same ICD-9 class (e.g. cancers) occupy the same local network neighborhood. To achieve this, we calculate mutual information between structural communities and ICD-9 class. Mutual information, *I(C;D)*, measures how much information the two variables share. We assign disease nodes to a community using the Louvain community detection algorithm and we assign ICD-9 codes as described above. The mutual information of structural community (*C*) and ICD-9 disease class (*D*) is defined as follows:

$$I(C;\,D)=\sum _{{\rm{c}}\in {C}}\,\sum _{{\rm{d}}\in D}p({\rm{C}},D){\log }_{2}\left(\frac{p({\rm{C}},D)}{p(C)p(D)}\right)$$

where *p(C,D)* is the joint probability distribution of *c* and *d*, and *p(C)* and *p(D)* are the marginal probability distributions of *C* and *D*, respectively. To compute *p(C,D), p(C)*, and *p(D)*, we construct a matrix *A* where each index *A*[*c*_*i*_, *d*_*i*_] represents the number of diseases that are both in structural community *c*_*i*_ and disease class *d*_*i*_.

Mutual information values are computed for disease projections in miRNA space and in gene space. As a negative control, we computed the average mutual information in 100 randomized disease projections. Each randomized projection is created by first shuffling node labels on the bipartite miRNA-disease network and then generating the disease projection.

## Supplementary information

Supplementary information.
