# Supplementary material for: Predicting miRNA-based disease-disease relationships through network diffusion on multi-omics biological data
Source: Sci Rep. 2020 May 26;10:8705. doi: 10.1038/s41598-020-65633-6 (PMC7251138; doi:10.1038/s41598-020-65633-6)
Supplement: Supplementary file 1 — Supplementary information. [file 41598_2020_65633_MOESM1_ESM.docx]

**Supplementary Information**

**Predicting miRNA-based disease-disease relationships through network diffusion on multi-omics biological data**

**Marissa Sumathipala^1,3^ *, Scott T. Weiss^1,2^**

^1 Channing Division of Network Medicine, Department of Medicine, Brigham and Women’s Hospital, Harvard Medical School, Boston, MA, USA.^

^2 Department of Medicine, Harvard Medical School, Boston, MA, USA^

^3 Harvard College, Cambridge, MA, USA^

*** Correspondence:**Marissa Sumathipala
sumathipalam@college.harvard.edu

**Pseudocode for MAP Algorithm**

**MAP**(*DG, PPI, MPI*)

**input:**

*DG*: disease-gene associations

*PPI*: protein-protein interaction network

*MPI*: miRNA-gene interaction network

**output:**

*M*: the set of ranked candidate miRNAs

*W* ← adjacency matrix of the union of *PPI* and *MPI,* column normalized

*N* ← number of nodes in *W*

*a* ← 0.7 // restart probability

*I* ← identity matrix with same dimensions of *W*

*P* ← inverse( *a*(I – (*1 *- a)*W)* // converged matrix for random walk

**for** **each** disease *d* **in** *DG* **do**

*p^0^* ← vector with size *N*, initialized to all zeros // probability vector for step 0

*G* ← genes associated with disease *d*

**for** **each** gene *g* **in** *G* **do**

*p_g_^0^* ← 1 / (number of elements in *G*)

**end for**

*p^∞^* ← *P * p^0^* // steady state probability vector

*M_d_* ← subset of *p^∞^* representing all miRNA nodes // ranked miRNAs for disease *d*

**end for**

**return** *M*
